# Assessment of chemical species of lead accumulated in tidemarks of human articular cartilage by X-ray absorption near-edge structure analysis

**DOI:** 10.1107/S0909049510052040

**Published:** 2011-01-20

**Authors:** Florian Meirer, Bernhard Pemmer, Giancarlo Pepponi, Norbert Zoeger, Peter Wobrauschek, Simone Sprio, Anna Tampieri, Joerg Goettlicher, Ralph Steininger, Stefan Mangold, Paul Roschger, Andrea Berzlanovich, Jochen G. Hofstaetter, Christina Streli

**Affiliations:** a Atominstitut, Vienna University of Technology, 1020 Wien, Austria; b MiNALab, CMM-Irst, Fondazione Bruno Kessler, Via Sommarive 18, 38123 Trento, Italy; c Istituto di Scienza e Tecnologia dei Materiali Ceramici CNR, Faenca, Italy; d Institute for Synchrotron Radiation, Karlsruhe Institute of Technology, Campus South, 76344 Eggenstein-Leopoldshafen, Germany; e Ludwig Boltzmann Institute of Osteology, Hanusch Hospital of WGKK and AUVA Trauma Centre Meidling, 4th Medical Department, Hanusch Hospital, Vienna, Austria; f Department of Forensic Medicine, Medical University of Vienna, A-1090 Vienna, Austria; g Department of Orthopaedics, Vienna General Hospital, Medical University of Vienna, A-1090 Vienna, Austria

**Keywords:** X-ray absorption spectroscopy, Pb *L*_3_-edge XANES, human bone, tidemark, trabecular bone

## Abstract

A highly specific accumulation of the toxic element lead was recently measured in the transition zone between non-calcified and calcified normal human articular cartilage. This transition zone, the so-called ‘tidemark’, is considered to be an active calcification front of great clinical importance. However, little is known about the mechanisms of accumulation and the chemical form of Pb in calcified cartilage and bone. Using spatially resolved X-ray absorption near-edge structure analysis (µ-XANES) at the Pb *L*_3_-edge, the chemical state of Pb in the osteochondral region was investigated. The feasibility of the µ-XANES set-up at the SUL-X beamline (ANKA synchrotron light source) was tested and confirmed by comparing XANES spectra of bulk Pb-reference compounds recorded at both the XAS and the SUL-X beamline at ANKA. The µ-XANES set-up was then used to investigate the tidemark region of human bone (two patella samples and one femoral head sample). The spectra recorded at the tidemark and at the trabecular bone were found to be highly correlated with the spectra of synthetic Pb-doped carbonated hydroxyapatite, suggesting that in both of these very different tissues Pb is incorporated into the hydroxyapatite structure.

## Abstract 2

Lead is a toxic trace element that shows a highly specific accumulation in the transition zone between calcified and non-calcified articular cartilage, the so-called ‘tidemark’. Excellent agreement has been found between XANES spectra of synthetic Pb-doped carbonated hydroxyapatite and spectra obtained in the tidemark region and trabecular bone of normal human samples, confirming that in both tissues Pb is incorporated into the hydroxyapatite crystal structure of bone. During this study the µ-XANES set-up at the SUL-X beamline at ANKA was tested and has proven to be well suited for speciation of lead in human mineralized tissue samples.

## 1. Introduction

Exposure to the toxic element lead is associated with chronic diseases of the nervous, hematopoietic, skeletal, renal and endocrine systems (Järup, 2003 ▶). Pb is predominantly stored in the human skeleton, where approximately 95% of the total body burden is present (Wittmers *et al.*, 1988 ▶). Bone is a composite material consisting of an organic component of the matrix, which is predominantly of type-I collagen molecules, which assemble in a regularly staggered manner to form fibrils of several hundred nanometres diameter. These collagen fibrils are impregnated with and surrounded by small nanocrystallite particles of carbonated apatite (Fratzl *et al.*, 2004 ▶).

The tibia and patella have been widely used in epidemiologic studies to determine bone Pb levels by *in vivo* *K*-line X-ray fluorescence (XRF). However, owing to the large information depth (∼2 cm) when considering Pb *K*-lines (75 keV photons) in *in vivo* XRF analysis, signals are detected from a large bone volume and lack spatial resolution.

In recent studies we investigated the spatial distribution of Pb in the osteochondral region of normal human joints (Zoeger *et al.*, 2006 ▶, 2008 ▶) using Pb *L*α fluorescence lines (10.55 keV photons). The osteochondral unit has a highly complex structure, which is designed to ensure a frictionless movement in articulating joints and to transfer loads from articular cartilage to underlying bone tissue. It is basically composed of articular cartilage, calcified cartilage and subchondral bone (Wong & Carter, 2003 ▶). The zone of calcified cartilage is about 100 µm thick and provides the tight bonding of articular cartilage to subchondral bone.

Using synchrotron-radiation-based micro-X-ray fluorescence analysis (µ-XRF) in confocal geometry we could show a highly specific accumulation of Pb in the transition zone between calcified and non-calcified articular cartilage, the so-called ‘tidemark’ (Zoeger *et al.*, 2006 ▶, 2008 ▶). Pb levels in the tidemark were approximately 13-fold higher than in the subchondral and trabecular bone. The tidemark is considered to be a metabolically active mineralization front (Lemperg, 1971 ▶). However, very little is known about the process of mineralization in this region and even less about the mechanism of Pb accumulation. Moreover, surprisingly little is known about the chemical species of Pb even in calcified articular cartilage. Articular cartilage is predominantly type-II collagen and a non-collagenous matrix consisting mainly of proteoglycans and water as well as poorly crystalline hydroxy­apatite in the calcified cartilage region (Maroudas, 1979 ▶). A major component of proteoglycans is sulfur in the form of sulfate, binding large amounts of water to the cartilage tissue. Since the content of proteoglycan is not uniform, sulfur is also not homogeneously distributed in articular cartilage. The sulfur content reaches its maximum adjacent to the tidemark where it rapidly drops off (Reinert *et al.*, 2002 ▶). As Pb is a chalcogenic element, Pb is most likely bound to SH groups in bone areas showing high sulfur concentrations. However, the fact that sulfur reaches its maximum adjacent to the tidemark where it rapidly drops off suggests another mechanism of Pb incorporation into calcified bone. Calcium hydroxyapatite (CaHA) is known for its facile ion-exchange properties, playing a multifaceted role mainly in environmental and chemical applications (Ellis *et al.*, 2006 ▶; Chartier *et al.*, 2001 ▶; Ma *et al.*, 1993 ▶; Mavropoulos *et al.*, 2002 ▶; Meis *et al.*, 2000 ▶; Park *et al.*, 2002 ▶; Xu & Schwartz, 1994 ▶). CaHA has the ability to trap and store toxic heavy metals by replacing Ca^2+^ ions in hydroxyapatite with metal ions, for instance lead, cadmium copper or zinc. This mechanism is successfully used for *in situ* immobilization and remediation of toxic heavy metals in contaminated soil from landfills and contaminated ground- and waste water (Chaturvedi *et al.*, 2006 ▶; Ma, 1996 ▶; Ma *et al.*, 1994 ▶; Takeuchi & Arai, 1990 ▶). Therefore, the replacement of Ca ions in hydroxyapatite with Pb seems to be the most likely mechanism of binding lead in the calcified cartilage region. However, the different chemical composition of articular cartilage (*e.g.* high S content dropping off at the tidemark) could allow for the presence of various Pb compounds, such as sulfates, sulfides, carbonates and oxides.

To determine the species of Pb in the tidemark and bone we applied spatially resolved X-ray absorption spectroscopy (XAS) (Koningsberger & Prins, 1987 ▶; Stöhr, 1992 ▶; Proost *et al.*, 2003 ▶) at the *L*_3_ absorption edge of Pb (13.035 keV). The X-ray absorption near-edge structure (XANES) spectra were compared with those of various reference compounds. Spatial resolved (µ)-XANES measurements have been performed at the X-ray beamline of the Synchrotron Radiation Laboratory for Environmental Studies (SUL-X) of the synchrotron radiation source ANKA (Institute for Synchrotron Radiation, Karlsruhe Institute of Technology, Germany). Additionally, some reference compounds were analysed at the XAS beamline (unfocused beam) at ANKA confirming the reliability of the µ-XANES measurements.

## 2. Materials and methods

### 2.1. Bone samples

**Figure 1:**
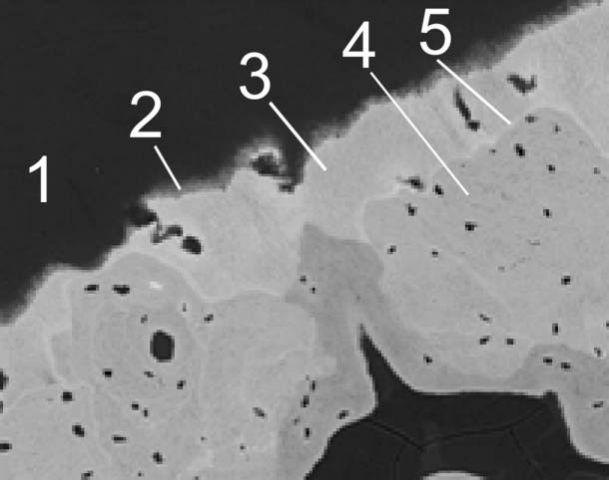
Backscattered electron image of human patella sample G3776. Articular cartilage (1), tidemark (2), calcified cartilage (3), subchondral bone (4), cement lines (5).

In this study two human patella samples previously investigated by confocal µ-XRF (Zoeger *et al.*, 2006 ▶, 2008 ▶) and one additional femoral head sample were analyzed using µ-XANES spectroscopy (Table 1 ▶). The samples were taken from randomly selected forensic autopsies of patients who died of an acute illness with no history of metabolic bone disease or Pb exposure. The selected samples showed no macroscopic signs of osteoarthritis. The study was approved by the Institutional Ethical Review Board of the Medical University of Vienna, Vienna, Austria. A 5 mm-thick section was cut perpendicular to the articular surface from the central region of the patella (sagittal plane). The sample was fixed in 70% ethanol, dehydrated through a series of alcohols, and embedded in polymethylmethacrylate (PMMA). The surfaces of the PMMA block were polished with diamond suspension and carbon coated for backscattered electron imaging (qBEI) as described previously (Roschger *et al.*, 1995 ▶, 1998 ▶). Sample slices of thickness 200 µm containing the bone area previously analyzed by qBEI imaging (see Fig. 1 ▶) were cut using a low-speed diamond saw (Buehler Isomed, Lake Pluff, USA).

### 2.2. Reference samples

Fourteen different Pb reference compounds have been investigated as being possible candidates for Pb compounds in bone: PbCO_3_ (Merck), PbSO_4_, PbO, PbO_2_, PbCl_2_, PbCO_3_·Pb(OH)_2_ (Fluka Chemie AG), PbS (Alfa Aesar), Pb(NO_3_)_2_, Pb(OH)_2_ as well as synthetic Pb-doped HA (hydroxyapatite) and CHA (carbonated hydroxyapatite) (Tabel 1 ▶). All reference materials were milled, mixed with polyethylene (Uvasol, Merck) or cellulose and pressed into pellets (pressure: 36.95 × 10^5^ Pa; time: 5 min). The HA powder standards were pressed with no additive, except Pb-HA 303 µg g^−1^ Pb, where 23% cellulose had been added.

The synthesis of Pb-substituted HA powders was carried out by neutralization of a calcium hydroxide suspension [Ca(OH)_2_] with an orthophosphoric acid solution (H_3_PO_4_) and adding lead nitrate [Pb(NO_3_)_2_] in the basic suspension in different amounts. In this way lead-substituted HA can be obtained, covering the entire range of substitution (Bigi *et al.*, 1989 ▶; Brückner *et al.*, 1995 ▶), where Pb replaces Ca in both octahedral and tetrahedral sites.

In order to assess whether the neutralization method allowed partially substituted HA to be obtained without any secondary phases containing lead, a synthesis of HA powder was performed, introducing 20 mol% of Pb, so to clearly put in evidence both the formation of secondary phases and the cell distortion owing to the substitution of calcium with lead.

X-ray diffraction analysis showed that the grown HA phase did not contain any additional phase; the analysis of element concentration, carried out on the Pb-HA powder by scanning-electron-microscope energy-dispersive spectroscopy (SEM-EDS), confirmed the presence of Pb in the product. The substitution of Ca with Pb (in the synthesis procedure with 20 mol% Pb) was also confirmed by Rietveld analysis, carried out using *TOPAS4.2* software, confirming the distortion of the hexagonal HA cell. In fact, the crystallographic axes of the HA cell are: *a* = 9.422 Å and *c* = 6.881 Å, and, in Pb-substituted HA, 9.545 Å and 6.934 Å, respectively. The increase in the lattice parameters is due to the replacement of Ca by the larger Pb ion.

The products of the HA syntheses were analyzed by inductively coupled plasma mass spectrometry (ICP-MS). The results are given in Table 2 ▶. All analyses show Pb in the HA powders; however, only a fraction of the introduced lead content was found in the products, while the rest was eliminated through washing out unreacted Pb(NO_3_)_2_ starting material. Details of the preparation of the synthetic Pb-doped HA references substances can be found in the supplementary material.^1^

### 2.3. Pb *L*_3_-edge XANES

XANES spectra are recorded by tuning the energy of incident X-ray photons across an absorption edge of a specific element present in the sample (here, Pb at the *L*_3_-edge). The XANES contains information about the chemical state of the absorbing element. One common method (fingerprinting) is to compare spectra of unknown samples (here, tidemark and subchondral bone) with those of reference compounds. Bulk XAS methods can be extended to µ-XANES (Proost *et al*., 2003 ▶) providing spatial resolutions in the micrometre range. Therefore, µ-XANES spectroscopy enables spatial determination of chemical species (*e.g.* to distinguish the species of Pb between tidemark and subchondral bone). In this study, recording full maps of chemical bindings was not feasible owing to the samples’ low Pb concentrations of about 5 µg g^−1^ (Rosen *et al*., 1993 ▶) to 10 µg g^−1^ (Somervaille *et al.*, 1985 ▶), causing extremely long measuring times for area scans. Hence, Pb *L*_3_-edge XANES spectra have been recorded at several representative points on the sample, selected from the results of Pb *L*α emission line scans.

Pb *L*_3_-edge XANES spectra of the bone samples and references listed in Table 1 ▶ were recorded at the SUL-X beamline in fluorescence mode. The synthetic Pb-HA samples were also measured at the ANKA-XAS beamline in fluorescence mode to compare the data quality of both beamlines for spectra from samples with low Pb concentrations.

**Figure 2:**
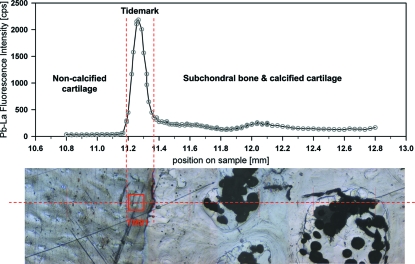
Pb *L*_3_ fluorescence intensities of a line scan across the tidemark of the bone sample G3776 correlated with the reflected light micrograph of the sample surface. The positions TM1 indicate the positions where XANES scans were performed [spot size, 150 µm (horizontal) × 100 µm (vertical)].

The X-ray source of SUL-X is a 27-pole wiggler operating at *K* = 8.5 (wiggler gap, 16 mm). A silicon (111) crystal pair with a fixed beam exit was used as monochromator. The X-ray beam was aligned with an intermediate focus, and finally focused by Kirkpatrick–Baez mirrors to about 100 µm (horizontal) × 20 µm (vertical) for measurements at the tidemark and to about 200 µm (horizontal) × 150 µm (vertical) for data acquisition in the trabecular bone. A linear scan across the calcification front of the bone sample was performed to determine the exact position for the XANES measurements at the tidemark. The Pb *L*α fluorescence intensity was evaluated for each step of the scan to find the maximum intensity which is known to coincide with the tidemark (Zoeger *et al.*, 2006 ▶, 2008 ▶). Fig. 2 ▶ shows an optical micrograph of the analyzed patella (sample G3776; below) and the Pb *L*α fluorescence intensity (above) recorded along the horizontal dashed red line. The marker TM1 indicates the positions where XANES scans were performed.

The fluorescence radiation was collected using a seven-element Si(Li) solid-state detector (Gresham, now e2v) and a digital signal-processing system (DXP) from XIA.

Three to nine scans were collected for both sample and reference substances. An energy calibration was performed and monitored using a Pb foil at the *L*_3_-edge in transmission mode.

The X-ray source of the ANKA XAS beamline is a 1.5 T bending magnet. The fixed-exit monochromator of the XAS beamline was operated in step-by-step mode using the Si 〈111〉 crystal pair. A five-element Ge solid-state detector (Canberra) was used to collect the fluorescence signal. At least two spectra for each reference sample were recorded. Similar to the SUL-X beamline the correctness of the energy calibration was monitored using a Pb foil at the *L*_3_-edge in transmission. No significant change could be detected.

For more details see the supplementary material.

### 2.4. XANES data analysis

The absorption spectra were recorded summing all fluorescence emission counts within the Pb *L*α region of interest. The resulting XANES spectra were analyzed using *ATHENA*, which is included in the *IFEFFIT* package for XAS analysis (Newville, 2001 ▶; Ravel & Newville, 2005 ▶; Newville, 2009 ▶). Each scan was normalized to an edge jump of unity and its energy scale was corrected with respect to the Pb *L*_3_-edge. Repetitive scans have been merged together to improve the signal-to-noise ratio.

## 3. Results

### 3.1. XANES spectra of Pb references

**Figure 3:**
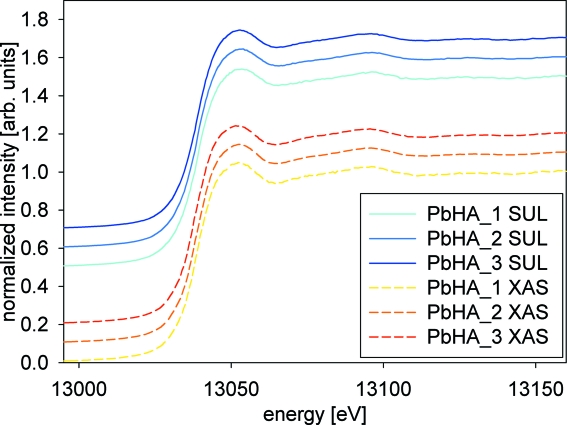
Comparison of the XANES spectra recorded for the Pb-HA reference compounds with different concentrations of Pb measured at the SUL-X and ANKA XAS beamlines.

The XANES spectra of the three Pb-HA reference compounds showing different Pb concentrations measured at the SUL-X and ANKA XAS beamlines are displayed in Fig. 3 ▶. Both beamlines produced identical results. At SUL-X the beam size at the sample position was about 200 µm (horizontal) × 150 µm (vertical) and at ANKA XAS 7 mm (horizontal) × 1 mm (vertical). The photon flux per 100 µm × 100 µm area on the sample at 13 keV was about 1 × 10^11^ photons s^−1^ at SUL-X and about 7.5 × 10^7^ photons s^−1^ at XAS. The photon flux at the sample at the SUL-X beamline was only slightly higher (about 3 × 10^11^ photons s^−1^) than at the ANKA XAS beamline (4 × 10^10^ photons s^−1^), explaining the similar quality of the spectra for both beamlines.

### 3.2. XANES spectra of Pb in bone

**Figure 4:**
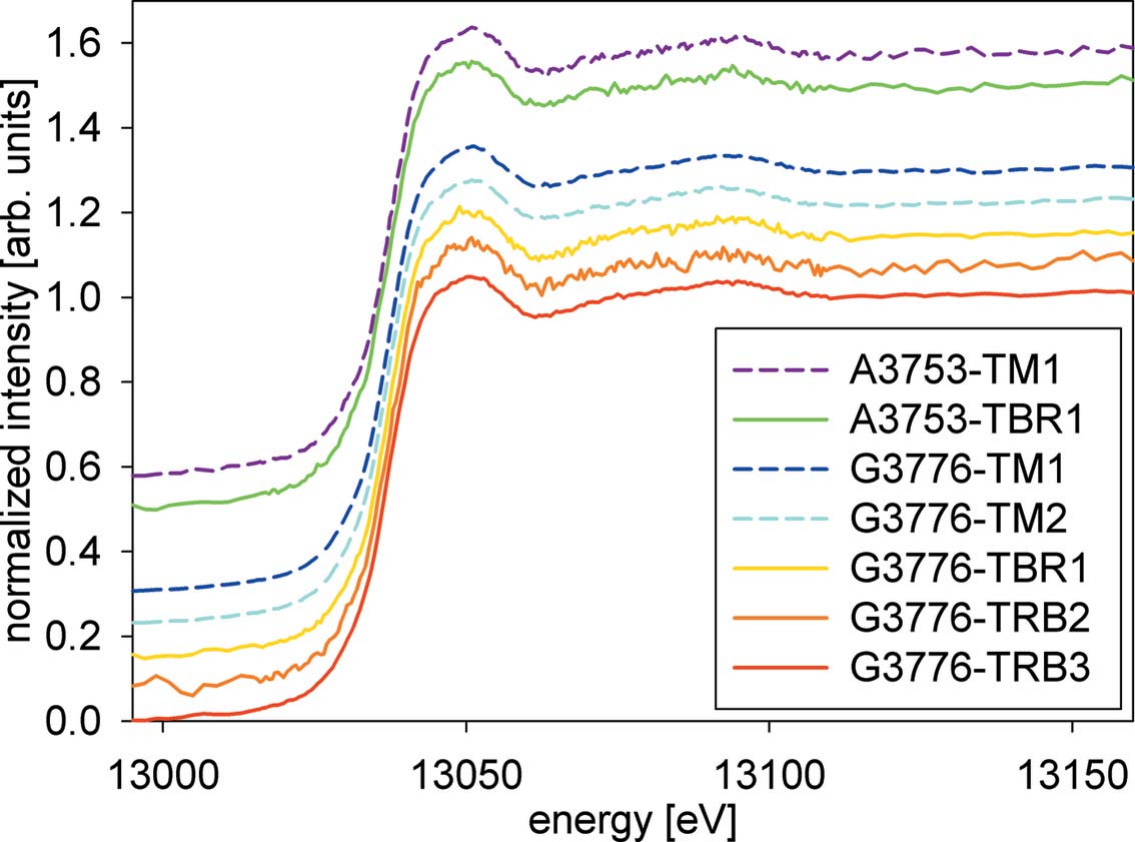
Comparison of XANES spectra of tidemark (TM) and trabecular bone (TBR) positions: A3753 femoral head, G3776 patella. The spectra are displaced vertically for clarity.

The XANES spectra recorded at three different positions (500 µm apart) at the tidemark (TM) of patella sample k3807 (data not shown), obtained at SUL-X, showed no differences in their features. The same result was obtained for different positions at the tidemark of patella sample G3776 and femoral head A3753. Moreover, there were no differences in the spectra between tidemark and trabecular bone position. Hence, all spectra from the bone samples have been merged to obtain a better signal-to-noise ratio for comparison with spectra of reference materials (Fig. 4 ▶).

### 3.3. Comparison of XANES spectra: tidemark/bone *versus* reference

**Figure 5:**
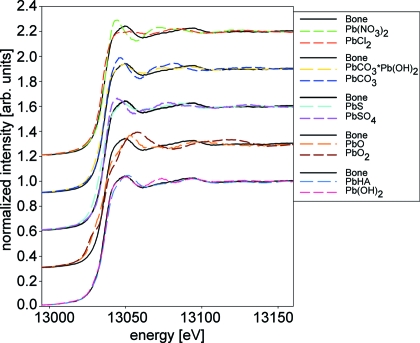
Pb *L*_3_-edge XANES spectra for all reference substances of this study compared with the merged XANES spectrum of all three bone samples (Bone).

**Figure 6:**
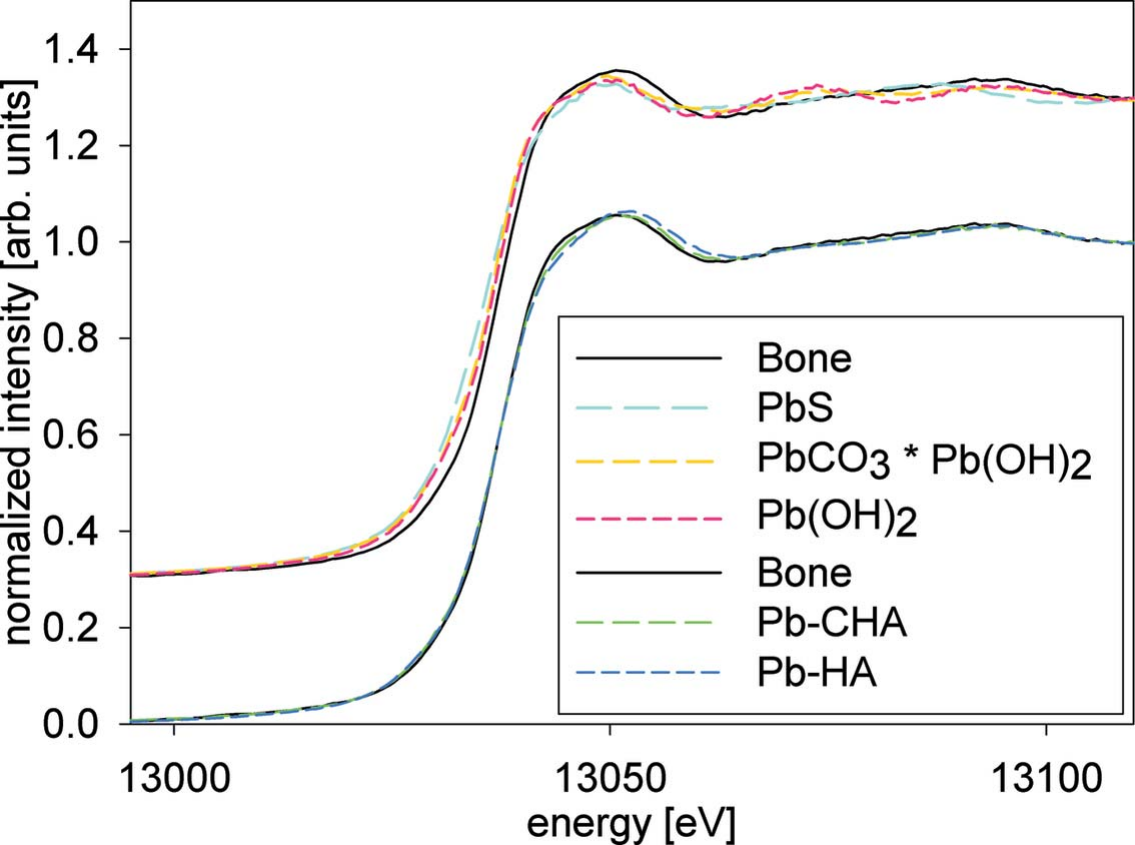
Comparison of best-fitting reference XANES spectra with a merge of all XANES spectra recorded for the different bone samples (Bone). The synthetic hydroxiapatite reference Pb-CHA has higher carbon content than the synthetic hydroxiapatite reference Pb-HA.

XANES spectra of the measured Pb reference compounds, including the synthetic Pb-HAs, are compared with the merged bone XANES spectrum in Fig. 5 ▶. It can be seen that spectra of five reference substances show similarities with the XANES recorded for bone. These are PbCO_3_·Pb(OH)_2_, Pb(OH)_2_, PbS, Pb-HA and Pb-CHA. For better clarity these spectra are displayed separately in Fig. 6 ▶.

**Figure 7:**
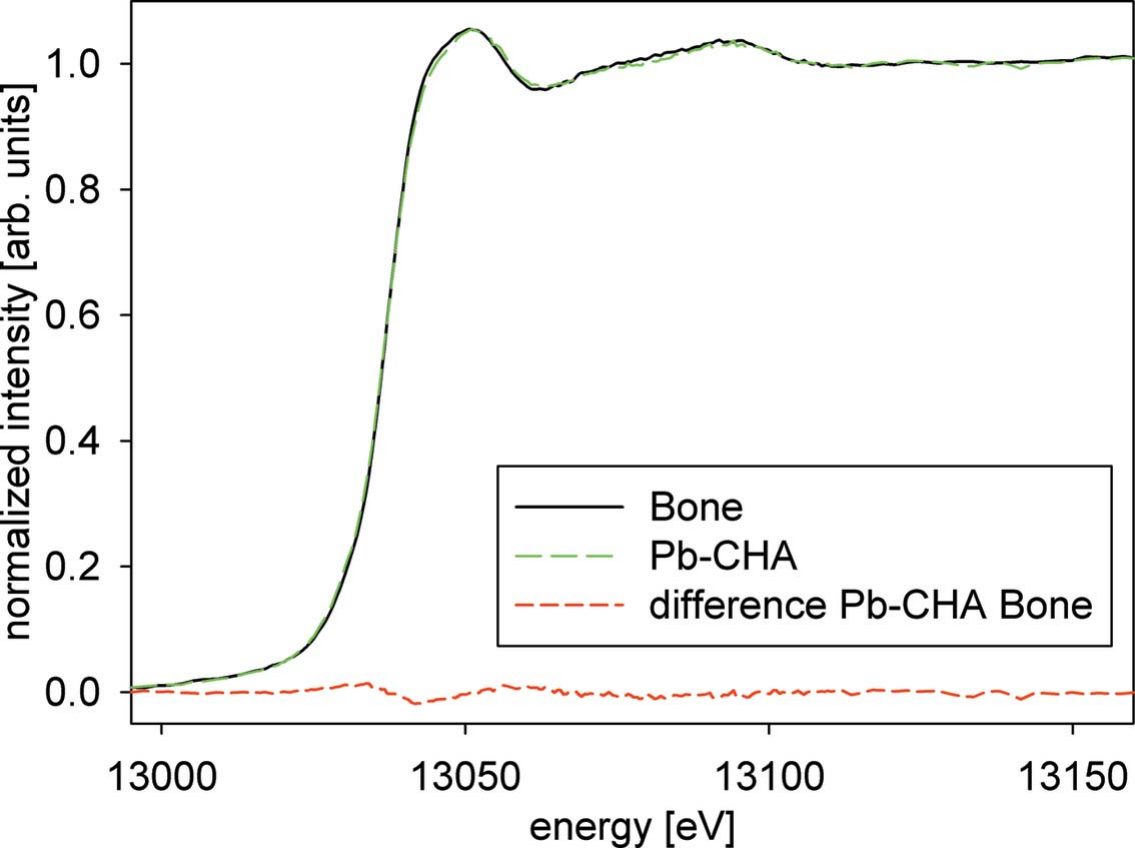
Pb *L*_3_-edge XANES spectra of merged tidemark/bone (Bone) spectra and synthetic carbonated Pb-CHA. The bottom (dotted red) line shows the difference between the two XANES spectra.

The best agreement with the XANES spectra from the bone samples was found for the synthetic Pb-substituted carbonated hydroxyapatite (Pb-CHA). These two XANES spectra are displayed in Fig. 7 ▶ along with their difference spectra. The residual sum of squares of the difference is about 0.01, proving the excellent match of the Pb-CHA reference material with the bone data.

## 4. Discussion

This study demonstrates that the spatial resolved µ-XANES technique on the SUL-X beamline is well suited to allocate the chemical form of Pb, present in only low concentrations in human bone and calcified articular cartilage tissue.

The feasibility of µ-XANES measurements at the Pb *L*_3_-edge of samples showing low Pb concentrations using the SUL-X beamline could be affirmed by comparing spectra from synthetic Pb reference compounds recorded at the SUL-X and the ANKA XAS beamlines, respectively. This is worth mentioning because the Pb concentrations in these reference compounds were rather low (∼300–1200 µg g^−1^ Pb; see Table 2 ▶). Although the number of photons hitting the sample area is slightly higher at the SUL-X beamline than at the ANKA XAS beamline it was not expected that the quality of the spectra of the SUL-X beamline would be equal or even slightly better. The higher flux of the SUL-X beamline seems to compensate for better averaging on the sample and being less sensitive to fluctuation of intensity and beam positions of the non-focusing optics at the ANKA-XAS beamline. The experimental results proved the competitive capability of the SUL-X µ-XANES set-up in comparison with a beamline dedicated to bulk XAS and assured the quality of the data from bone samples. The variations found in the XANES of reference compounds with Pb in different chemical environment demonstrated its sensitivity to allocate the chemical state of Pb in the samples.

The µ-XANES spectra recorded at different positions of the tidemark of both samples showed no differences, indicating that Pb is generally in the same chemical state at the tidemark of bone. However, it is most striking that no differences in the µ-XANES spectra between tidemark (calcification front) and trabecular bone region could be found, though the chemical environment/composition between both tissue regions are known to be very different (Kuhlman, 1980 ▶). No other intermediate mechanisms of binding seem to take place at the calcification front of articular cartilage. The comparison of the µ-XANES spectra of bone with that of the reference compounds suggests that Pb is incorporated into bone by replacing Ca^2+^ ions in HA as found in bone and tidemark.

The substitution of Pb in CaHA is a well known mechanism in the fields of biochemistry and environmental science, where synthetic HA is successfully applied in remediation of heavy-metal-contaminated soil, water or waste (Chaturvedi *et al.*, 2006 ▶). Besides lead, metal substitution on the Ca site involves, for example, the biological important iron, copper, zinc and cadmium. The Pb^2+^ in solution exchanges with Ca, causing morphological changes in the surface region. In the case of high Pb concentrations the formation of Pb-substituted CaHA is followed by the formation of the pyromorphite phase, where all Ca is replaced with lead (Ma *et al.*, 1993 ▶; Xu & Schwartz, 1994 ▶). Currently, four possible immobilization routines including the ion exchange process, surface complexation, dissolution and precipitation and co-precipitation have been generally suggested for the Pb immobilization mechanism of HA (Ellis *et al.*, 2006 ▶).

The mechanism of lead substitution in the apatite structure has been studied at high Pb concentrations. According to Bigi *et al.* (1989 ▶) and Brückner *et al.* (1995 ▶) it can be assumed that the same is true for very low Pb concentrations (for example, in the Pb-doped synthetic apatites and in the bones samples). Therefore, it can be excluded that separate Pb-containing phases are present, leading to the conclusion that Pb in bones is in fact incorporated into the apatite structure.

Among the investigated Pb reference compounds, Pb-CHA was found to fit best to the spectra recorded at tidemarks and trabecular bone tissue. This is in agreement with the fact that carbonate substitution occurs at both hydroxyl and phosphate sites, and, along with Ca-deficient HA, plays an important role in the nanocrystalline structure of bone tissue (Ellis *et al.*, 2006 ▶). Bone is a composite material containing mineral nanocrystallites and a protein matrix that is predominantly cross-linked type-I collagen. Previous studies have shown that the mineral is a carbonated calcium phosphate within the apatite crystalline structure group. Moreover, bone is unique in comparison with the geological apatite HA in that carbonate substitutes for about 68% of the phosphate and relatively little hydroxide is present (20% or less) (Awonusi *et al.*, 2007 ▶; Cho *et al.*, 2003 ▶; Pasteris *et al.*, 2004 ▶).

The tiny differences observed in µ-XANES between tidemark/bone and the Pb-CHA reference right above the absorption edge suggest either that still a very small fraction of Pb is present in an unknown state or that the carbon concentration in the HA reference material does not perfectly match the carbon content in human bone material.

While most of the health hazards of lead are well known (Järup, 2003 ▶), the effects of Pb on cartilage metabolism are less well studied. Chondrozytes seem to be important target cells for the toxic effects of Pb (Puzas *et al.*, 1992 ▶). Intra-articular lead has been shown to lead to osteoarthritic changes in the knee joint in humans (Slavin *et al.*, 1988 ▶) as well as in experimental animal models (Harding *et al.*, 1999 ▶; Bolanos *et al.*, 1995 ▶). Moreover, it was shown in an experimental mouse model that exposure to lead inhibits fracture healing. It was possible to show that Pb exposure leads to an increased cartilage formation with delayed maturation and calcification and increased formation of fibrous tissue during bone repair (Carmouche *et al.*, 2005 ▶). However, at this stage, it is still unclear what the medical consequences of Pb accumulation in the tidemark region are.

In conclusion, the µ-XANES set-up at the SUL-X beamline at ANKA has proven to be well suited for the determination of the species of lead in human bone and cartilage samples. The outcome, that Pb is bound to carbonated hydroxyapatite in the tidemark, sheds new light onto the specific accumulation on Pb in this sensitive articular joint. Previously suggested mechanisms for immobilization of Pb in HA (Ellis *et al.*, 2006 ▶) may be involved in the incorporation of lead into calcified bone tissue and the tidemark.      

## Supplementary Material

Supplementary material file. DOI: 10.1107/S0909049510052040/hf5181sup1.pdf

## Figures and Tables

**Table:** 

Sample	Tidemark	Trabecular bone
Human patella k3807	k3807_Pos1	Not measured
	k3807_Pos2	
	k3807_Pos3	
Human patella G3776	G3776_TM1	G3776_TBR1
	G3776_TM2	G3776_TBR2
		G3776_TBR3
Human femoral head A3753	A3753_TM1	A3753_TBR1

**Table:** 

Reference materials	
PbCO_3_	Pb(OH)_2_
PbSO_4_	PbCO_3_·Pb(OH)_2_
PbO	Pb-HA_1 (400 µg g^−1^ Pb)
PbS	Pb-HA_2 (700 µg g^−1^ Pb)
PbCl_2_	Pb-HA_3 (1200 µg g^−1^ Pb)
Pb(NO_3_)_2_	Pb-HA (303 µg g^−1^ Pb)
PbO_2_	Pb-CHA (335 µg g^−1^ Pb)

**Table 2:** Chemical analysis of Pb-substituted HA powders

	Expected molar Pb/Ca	Actual molar Pb/Ca	Pb content (µg g^−1^)
Pb-HA_1	0.001	0.0004	400
Pb-HA_2	0.005	0.0035	700
Pb-HA_3	0.01	0.0077	1200
Pb-HA	0.0005	0.0002	303
Pb-CHA	0.0005	0.0002	335

## Footnotes

1 Supplementary data for this paper are available from the IUCr electronic archives (Reference: HF5181). Services for accessing these data are described at the back of the journal.
