# Supplementary material for: Assessment of chemical species of lead accumulated in tidemarks of human articular cartilage by X-ray absorption near-edge structure analysis
Source: J Synchrotron Radiat. 2011 Jan 20;18(Pt 2):238–44. doi: 10.1107/S0909049510052040 (PMC3042330; doi:10.1107/S0909049510052040)
Supplement: Supplementary file 1 [file s-18-00238-sup1.pdf]

Synthesis of Pb-substituted hydroxyapatites

The synthesis of Pb-substituted HA powders was carried out by neutralization of a calcium hydroxide suspension ($Ca(OH)_2$, 95% pure) with a orthophosphoric acid solution (H_3PO_4, 85% pure). Lead nitrate ($Pb(NO_3)$2, 99.5% pure) was added in the basic suspension and in different amounts, so to have starting Pb/Ca molar ratios of 1, 0.5, 0.1, and 0.05 mol%, according with the general formula $Ca_{10-x}Pbx(PO_4)_6(OH)_2$. All HA powders contain some carbonate due to spontaneous carbonation of the reaction with CO_2 dissolved in water. One reference with Pb/Ca 0.05 mol% was additionally carbonated; all reactants are provided by Sigma-Aldrich (S. Louis, MO, USA). The Pb-HA powders were synthesized in a Pyrex flask at ambient temperature (22-23 °C) by using 0.38 mol of $Ca(OH)_2$ suspended in 0.7 l of H_2O: the acid solution was dropped in the stirring basic suspension and, after, the powders were ripened in the mother liquor for 24 hours, then washed in 2 liters of distilled water and centrifuged for three times, prior drying at 40 °C in ventilated oven and sieving at 150 mm.

The phase composition of the as obtained HA powders was determined by X-ray diffraction (XRD: Cu K_α radiation, Miniflex Rigaku, Tokyo, Japan); the chemical composition of the powders, namely the content of calcium, phosphorous and lead, was evaluated by inductively coupled plasma-optical emission spectrometry (ICP-OES: Liberty 200, Varian, Clayton South, Australia) and energy dispersive spectroscopy (EDS: INCA 300, Oxford Instruments, UK) with a scanning electron microscopy system (SEM: Stereoscan 360, Leica, Cambridge, UK).

Five different HA powders, to be used a references for the XANES measurements, were obtained and analysed: PbHA_1, PbHA_2 PbHA_3, Pb-HA and Pb-CHA characterized by a starting molar Pb/Ca ratio of 0.1, 0.5, 1, and 0.05 mol% respectively. Figure 1 shows the X-ray diffractograms of the first three obtained powders: The only crystalline phase detected is hydroxyapatite of very low crystallinity, indicated by the broadening of the diffraction peaks.

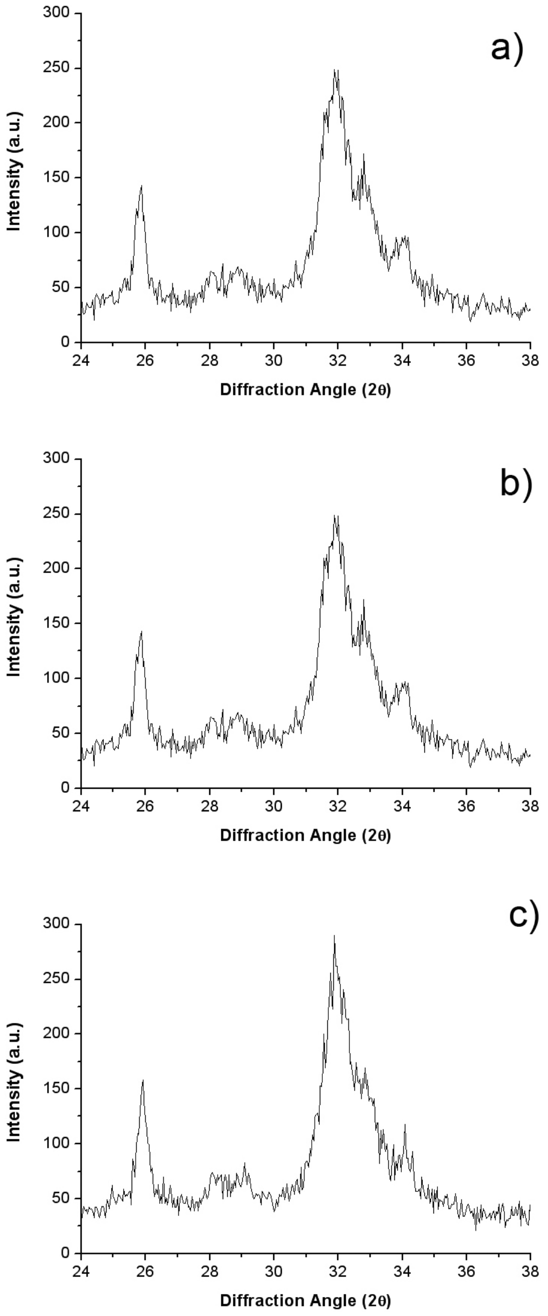

Figure 1 X-ray diffractograms of Pb-HA powders: a): PbHA_1; b): PbHA_2; c): PbHA_3 showing the (002) reflexes (at about 25.87 ° 2 Theta) and the (211), (112) and (300) reflexes (between about 31 ° and 33 ° 2 Theta) which are not angular resolved because of the peak broadening due to small crystallite sizes.

Setup and Parameters for the Pb L_3-edge XANES Measurements at the ANKA SUL-X and XAS Beamlines

The X-ray source of SUL-X is a 27 pole Wiggler operating at K=8.5 (wiggler gap 16 mm). A silicon (111) crystal pair with a fixed beam exit was used as monochromator. The X-ray beam was aligned to an intermediate focus, and finally focused by Kirckpatrick-Baez mirrors to about 100 µm (hor.) x 20 µm (vert.) for measurements at the tidemark and to about 200 µm (hor) x 150 µm (vert.) for data acquisition in the trabecular bone.

Pressed pellets of various Pb-compounds and synthetic Pb doped hydroxyapatite were measured as references. Transmission data was used for the Pb reference compounds (except the synthetic apatites) because fluorescence data for highly concentrated standards might be affected by self absorption effects. For the synthetic apatites and the bone samples fluorescence acquisition was chosen since concentrations of Pb are in the range below 1 wt %. The energy window was set to the Pb L_α lines. The fluorescence radiation was collected with a 7 element Si(Li) solid state detector (Gresham, now e2v) and a digital signal processing system (DXP) from XIA. Three up to nine scans (1-1.5 h each) were collected for both, sample and reference substances, with 3 or 5 s fixed accumulation time per energy step up to 20 or 75 eV above E_0; above these energies the scan was continued using k steps with increasing accumulation time according to time*$k^{0.5}$. Data were dead time corrected, summed up for all seven channels and divided by the input intensity measured with an ionization chamber prior to the sample (Oxford IC-Plus). Energy calibration was performed and monitored with a Pb foil at the L_3 edge in transmission mode and was found to be unaffected during the scans.

The synthetic Pb-HA samples were also measured at the ANKA-XAS beamline in transmission and fluorescence mode to compare the data quality of both beamlines for spectra from samples with low Pb concentrations. The X-ray source of the ANKA XAS beamline is a 1.5 Tesla bending magnet. The fixed exit monochromator of the XAS beamline was operated in step by step mode using the Si<111> crystal pair. Due suppress the higher harmonics a detuning to 70 % of the maximum beam intensity by a D-Mostab (Struck, Hamburg) was used. Three ionization chambers of the type Oxford X-Spec were used to acquire the transmission signal of the sample and the metallic lead foil. A five element Ge solid state detector (Canberra) combined with the digital signal processing system (DXP) from XIA was used to collect the fluorescence signal. At least tow spectra for each reference sample were taken with a measuring time of 3-4 seconds before and up to 20 eV above the edge. Beyond 20 eV above the edge the measured energy point were chosen equidistance to the k-vector and the acquisition time were increased with a factor of $k^{0.5}$. Similar to SUL-X the correctness of the energy calibration was monitored by with a Pb foil at the L_3 edge in transmission. No significant change could be detected.
